# *All-Trans* Retinoic Acid-Induced Deficiency of the Wnt/β-Catenin Pathway Enhances Hepatic Carcinoma Stem Cell Differentiation

**DOI:** 10.1371/journal.pone.0143255

**Published:** 2015-11-16

**Authors:** Xinfeng Zhu, Wenxue Wang, Xia Zhang, Jianhua Bai, Gang Chen, Li Li, Meizhang Li

**Affiliations:** 1 Affiliated Calmette Hospital of Kunming Medical University, Kunming, Yunnan Province, 650011, P. R. China; 2 Laboratory of Biochemistry and Molecular Biology, School of Life Sciences, Yunnan University, Kunming, Yunnan Province, 650091, P. R. China; Laboratoire de Biologie du Développement de Villefranche-sur-Mer, FRANCE

## Abstract

Retinoic acid (RA) is an important biological signal that directly differentiates cells during embryonic development and tumorigenesis. However, the molecular mechanism of RA-mediated differentiation in hepatic cancer stem cells (hCSCs) is not well understood. In this study, we found that mRNA expressions of RA-biosynthesis-related dehydrogenases were highly expressed in hepatocellular carcinoma. *All-trans* retinoic acid (ATRA) differentiated hCSCs through inhibiting the function of β-catenin *in vitro*. ATRA also inhibited the function of PI3K-AKT and enhanced GSK-3β-dependent degradation of phosphorylated β-catenin. Furthermore, ATRA and β-catenin silencing both increased hCSC sensitivity to docetaxel treatment. Our results suggest that targeting β-catenin will provide extra benefits for ATRA-mediated treatment of hepatic cancer patients.

## Introduction

Cancer stem cells (CSCs) represent a small but unique population of cancer cells that can sustain their self-renewal and multipotentiality. These cells are also considered cancer-initiating cells (CICs) because their tumorigenic potency allows CSCs/CICs to form *de novo* tumors [1–2]. Clinical studies suggest that CSCs/CICs are resistant to traditional chemo- or radio-therapies due to their specialized microenvironments [3–4]. It has been proposed that both microenvironment-providing signals and their intracellular molecule transductions are essential for protecting and maintaining CSCs/CICs. Several important pathways such as Notch, Hedgehog and Wnt have been identified in the maintenance or differentiation of CSCs/CICs (5).

Liver cancer is one of the most common and lethal cancers worldwide [5–6]. Unfortunately, clinical therapies for liver cancer are still not available due to the heterogeneous nature of the cellular components within the tumors [5]. In hepatocellular carcinoma (HCC), cell membrane proteins such as CD133, CD13, CD90, EpCAM and CD44 have been widely applied to isolate different types of hepatic cancer stem cells (hCSCs) [7–13]. These hCSCs have demonstrated similar resistance to anti-cancer drugs [10,14].

Wnt/β-catenin has been traditionally considered the differentiation signaling pathway during embryonic development through its regulation of cell differentiation, proliferation and migration [15–20]. However, recent pluripotential stem cell studies have also suggested that Wnt/β-catenin may be essential to maintain their stemness [21–25]. In particular, β-catenin plays dual roles of regulator to enhance the undifferentiated status of embryonic stem cells [23–24]. Wnt/β-catenin signaling is reportedly involved in the survival and proliferation of normal and tumorigenic liver progenitor cells [25–28], and facilitates the stemness of cancer stem cells [29–31]. Clinical studies have suggested that activation of β-catenin is associated with the dedifferentiation of neoplastic hepatocytes to immature progenitor cells [32]. β-catenin function is regulated by the axin-APC-GSK-3 β-CK1α protein complex, which degrades β-catenin through protein phosphorylation [33].

Retinoic acid (RA) is another important differentiation signal for stem cells [34–35]. In the liver, RA biosynthesis is dependent on the dehydrogenases, including retinol dehydrogenases (RDH1, RDH2, RDH1), alcohol dehydrogenases (ADH1, ADH2, ADH3) and retinaldehyde dehydrogenases (RALDH1, RALDH2, RALDH3) [34]. Three retinoic acid receptors (RARα, RARβ and RARγ) have been identified as cognate receptors for RA. These RARs are structurally- and functionally-conserved nuclear retinoid receptors [36–39]. RA induces the differentiation of tumor progenitors and arrests their cell proliferation [40–41]. Two hypotheses have been suggested to explain the molecular mechanisms of RARs: First, activated RARs translocate from the cytoplasm to the nucleus and bind to RA-responsive elements to regulate down-stream gene transcriptions [42–43]. Second, RARs transiently activate the cytoplasmic kinase cascades, including p38MAPK, ERK and MAPKs [43–53]. Although *all-trans* retinoic acid (ATRA) is a clinically promising drug for the treatment of leukemia and solid cancers [42, 54–56], its detailed molecular mechanism remains little understood.

RA exposure can inhibit β-catenin function during early palate development [57] as well as tumor growth [58]. ATRA-induced inhibition of β-catenin function is related to the PI3K/AKT-dependent regulation of GSK3β [57,59]. GSK3β sequentially induces the protein degradation of phosphorylated β-catenin [60–62]. Given that ATRA and β-catenin both participate in the maintenance of cancer stem cells, characterizing the molecular relationship between RA and β-catenin in hCSCs is important to develop more effective therapies for HCC. In this study, we investigated ATRA-mediated differentiation and the potential roles of β-catenin during hCSC differentiation.

## Materials and Methods

### Cell culture

Cell lines (HepG2, Huh-7 and PLC-PRF-5) from human hepatic carcinoma were cultured in Dulbecco's modified eagle medium (DMEM, Gibco, China) supplemented with 10% fetal bovine serum (FBS, Biological Industries, Australia) in an incubator with 5% CO_2_ at 37°C. The CD133^+^ hCSCs from the HepG2 and PLC-PRF-5 cell lines were maintained with DMEM/F12 medium (Gibco, USA) containing 20 ng/ml EGF, 10 ng/ml FGF2 and 2% B27 (Miltenyi Biotec, Germany). The CD133^+^ cells-derived spheres were cultured in suspension cell culture plates (Beaver Bio, China). The CD133^-^ non-hCSC cells were cultured in DMEM (Gibco, China) with 10% FBS, as described above.

### Flow cytometry

Mouse anti-human CD133 antibodies conjugated with the fluorochromes of APC, PE or FITC were purchased from Miltenyi Biotec (Germany). Suspended cells were incubated by antibodies for 10 minutes at 4°C. The stained cells were further analyzed by an Accuri C6 flow cytometer (Becton Dickinson, USA). The APC-, PE- and FITC-conjugated mouse IgG1s were used synchronously as the isotype controls.

### Sorting CD133^+^ hCSCs

The CD133^+^ hCSCs were isolated using magnetic beads according to the standard procedure. In brief, cells were trypsinized to obtain an individual cell suspension and incubated with magnetic beads with anti-human CD133 antibodies (Miltenyi Biotec, Germany) for 30 minutes at 4°C. Cells were loaded onto LS columns and CD133^+^ cells were separated from other cells using a QuadroMACS^™^ Separator (Miltenyi Biotec, Germany). CD133^+^ cells were collected and suspended in DMEM/F12 media supplemented with 20 ng/ml EGF, 10 ng/ml FGF2 and 2% B27 (Miltenyi Biotec, Germany).

### Drug treatments

Stock concentrations of ATRA (Sigma, USA), 9-cis retinoic acid (Sigma, USA) and docetaxel (Sigma, USA) were prepared in dimethyl sulfoxide (DMSO). The three drugs were further diluted into DMSO to obtain different working concentrations.

### Western blot analysis

Rabbit anti-human CD133, β-catenin, NANOG, OCT4 and SOX2 antibodies were purchased from Huabio (China) for western blot analysis. Rabbit anti-human phospho-RARα (Ser77), RARβ and phospho-β-catenin (Tyr489) antibodies were bought from Anbo Biotechnology (China). Rabbit anti-human Akt, phospho-Akt (Thr308) and tubulin antibodies were purchased from Beyotime Biotechnology (China), and rabbit anti-human PI3K was bought from Antibody Revolution (USA). Western blots were conducted according to standard procedures.

### Immunohistochemistry

Human HCC specimens were obtained from the Department of Pathology of the Affiliated Calmette Hospital of Kunming Medical University. Immunohistochemical staining was conducted according to the standard procedures. Briefly, paraffin-embedded sections were incubated with antibodies of rabbit anti-human RARβ (Anbo Biotechnology Company, China), RALDH1 (Abgent, USA), RDH10 (Abgent, USA) and ADH1 (Beijing Biosynthesis Biotechnology Co., Ltd, China) overnight at 4°C. Sections were further stained with goat anti-human secondary antibodies conjugated with horseradish peroxidase (Santa Cruz Biotechnology, USA). Signals were developed with DAB reagent (Boster Biological Technology Ltd., China). Sections were examined under a light microscope (Olympus, Japan). Histological results were finally confirmed by a pathologist and with surgery conducted at the Affiliated Calmette Hospital of Kunming Medical University.

### qPCR

Total RNAs were purified from cells using RNAiso Reagent (TaKaRa, China), and reverse-transcription (RT) reactions were conducted to obtain cDNAs using a PrimeScriptTM RT Reagent Kit (TaKaRa, China). SYBR Primix Ex TaqTM (TaKaRa, China) was used for real-time fluorescence PCR (qPCR). Experiments were carried out using a real-time PCR machine (Applied Biosystems, USA).

### Cell proliferation assay

Cells were seeded into 96-well plates at a density of 500 cells per well. Next day, various doses of DOC and ATRA were used to treat the cells. Cell proliferation was analyzed by a CellTiter 96^®^ Aqueous One Solution Cell Proliferation Assay kit (Promega, USA). Cells were incubated for 2 hours, with cell proliferation then determined based on color change. We used a SpectraMax M2 (Molecular Devices, USA) to test OD values at 490 nm.

### Knockdown of β-catenin mRNA

β-catenin shRNA/PLKO.1 and PLKO.1 empty plasmids were purchased from Sigma (USA). To knockdown the mRNA expression level of β-catenin, two stably-interfered cell lines were established according to the standard experimental procedure. Briefly, β-catenin shRNA/PLKO.1 (or PLKO.1) plasmids together with pMD2.G and psPAX2 plasmids were mixed with FuGENE HD transfection reagent (Roche, Germany) and incubated at RT for 20 minutes, with the mixture then applied to the transfect 293T cells. Lentiviral particles were harvested 48 hours after transfection and added to the hCSCs for an extra 48 hours. Stable cancer stem cells were further screened with 10 μg/ml puromycin (Tocris, UK) for 15 days.

### Apoptotic assay

HepG2 CD133^+^ hCSCs were cultured in 6-well suspension culture plates and treated with different concentrations of ATRA (10^−6^ ~ 10^−8^ M) or DOC (10^−8^ ~ 10^−10^ M), or by a combination of ATRA (10^−6^ ~ 10^−8^ M) and DOC (10^−9^ M) for 48 hours. Cells were digested by 0.25% trypsin into a single cell suspension and incubated with 5 μl Annexin V-FITC and 5 μl propidium iodide (PI) solution (Apoptosis Detection kit, Vazyme Biotech Co., Ltd, China) for 10 minutes at RT. The apoptotic assay was conducted using flow cytometry (Accuri C6, Becton Dickinson, USA).

### Bioinformatics

The Oncomine (Compendia Bioscience; http://www.oncomine.org) database was used for bioinformatic analysis. Relevant analytical parameters are provided in the figures.

### Ethics committee approval

The Ethics Committee of the Affiliated Calmette Hospital of Kunming Medical University approved this study. Under this supervision, written informed consent from the donors or the next of kin was obtained for use of the sample in this research.

### Statistical analysis

All experiments were repeated three times. Statistical analyses were performed using SPSS 13.0 software. Cell proliferation and qPCR were assessed by One Way ANOVA.

## Results

### Retinoic acid metabolism was abnormally increased in liver cancers

**Fig 1:**
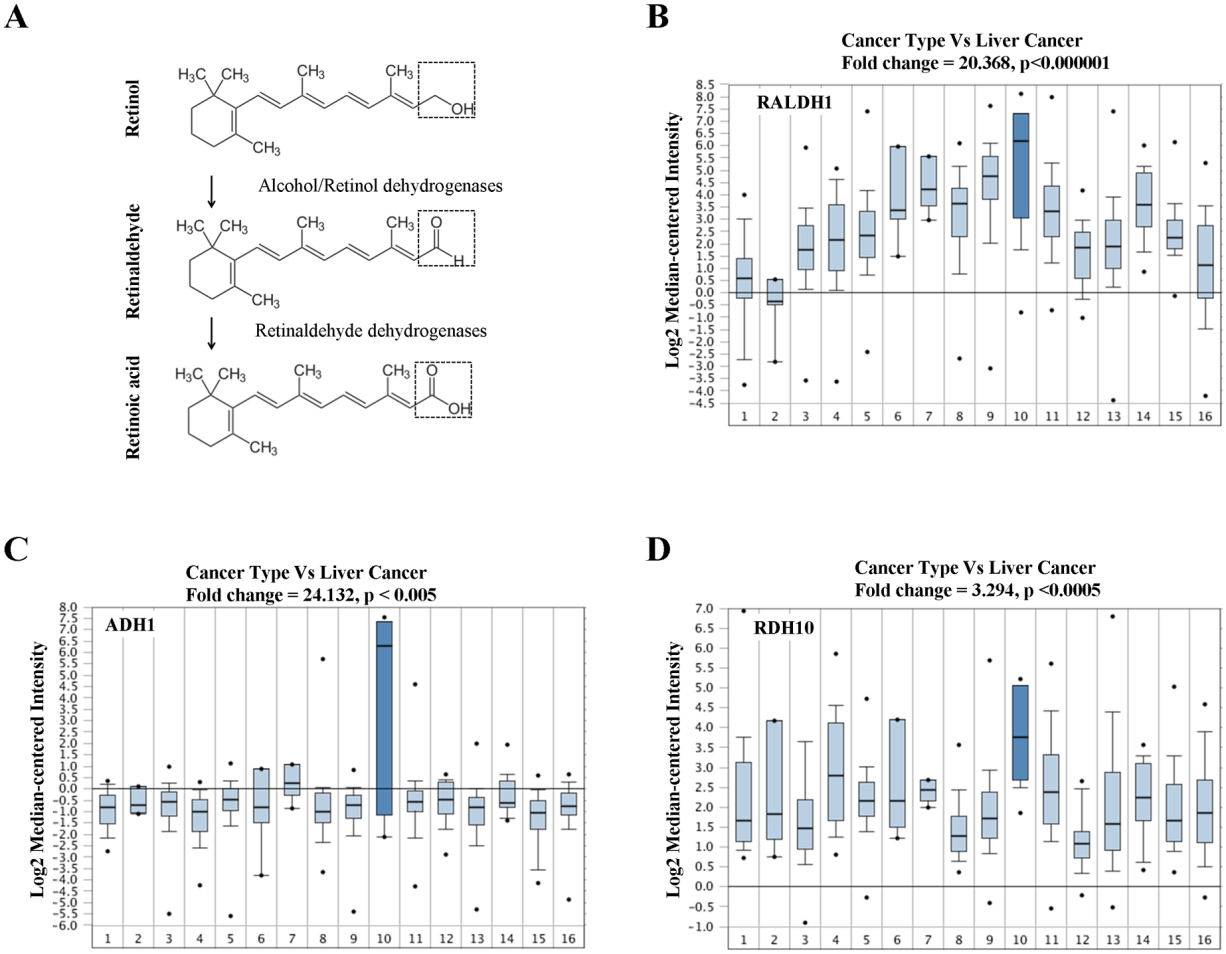
Gene profiling of dehydrogenases related to RA synthesis in different cancer types. **(A)** The process of RA synthesis and catalytic enzymes. Three alcohol dehydrogenases (ADH1, ADH3, ADH4), two retinol dehydrogenases (RDH1 and RDH10) and three retinaldehyde dehydrogenases (RALDH1, RALDH2, RALDH3) are involved in the biosynthesis of retinoic acid from retinol. The main catalyzing sites for chemical retinol, retinaldehyde and retinoic acid are highlighted by dashed boxes. **(B-D)** Gene expression profiling of RALDH1, ADH1 and RDH10 was analyzed using the Oncomine database (Bittner Multi-cancer data set, including 1,911 clinical patients, pathologically classified by 16 different cancer types). (**B**) RALDH1; (**C**) ADH1; (**D**) RDH10. Liver cancer (HCC) group is highlighted by dark blue. (1) Bladder cancer (n = 32); (2) Brain and central nervous system cancer (n = 4); (3) Breast cancer (n = 328); (4) Cervical cancer (n = 35); (5) Colorectal cancer (n = 330); (6) Esophageal cancer (n = 7); (7) Gastric cancer (n = 7); (8) Head and neck cancer (n = 41); (9) Kidney cancer (n = 254); (10) Liver cancer (n = 11); (11) Lung cancer (n = 107); (12) Lymphoma (n = 19); (13) Ovarian cancer (n = 166); (14) Pancreatic cancer (n = 19); (15) Prostate cancer (n = 59); (16) Sarcoma (n = 49).

The human liver is an actively RA-synthesizing organ. To address RA metabolism in HCC, we investigated three kinds of RA synthesis-related enzymes in HCC, including three alcohol dehydrogenases (ADH1, ADH2, ADH 3), two retinol dehydrogenases (RDH1, RDH10) and three retinaldehyde dehydrogenases (RALDH1, RALDH 2, RALDH 3) (Fig 1A). Patient-specific gene profiling was analyzed according to the online clinic liver cancer database (Oncomine: https://www.oncomine.org). Results from a total of 1,911 individual cancer patients demonstrated that the mRNA expressions of RALDH1, ADH1 and RDH10 were highest in HCC patients (Fig 1B–1D, dark blue histogram bars). Among a total of 16 different cancer types, the mRNA expression of RALDH1 showed a significant (p < 0.000001) 20-fold increase in HCC compared with that in other cancer types (Fig 1B). Similar results were obtained for mRNA expressions of ADH1 and RDH10 in HCC (Fig 1C and 1D). We compared the mRNA expressions of three rate-limiting enzymes, RALDH1, RALDH2 and RALDH3, between liver cancers and normal liver tissues, and found that the mRNA expressions of RALDH1 and RALDH2 were slightly but significantly increased in HCC compared with that in normal liver (S1A–S1D Fig). Our findings demonstrated that RA-related metabolizing activities were abnormally increased in HCC, suggesting that RA was an enriched signal provided by the local microenvironment of liver cancers.

### Binding of *all-trans* retinoic acid activated retinoic acid receptors in CD133^+^ hepatic cancer stem cells

To understand the role of RA signaling in CD133^+^ hCSCs, we first used flow cytometry to examine the percentages of CD133^+^ cells in different hepatic carcinoma cell lines (HepG2, Huh-7 and PLC-PRF-5). Interestingly, we found that about 11.7% of HepG2 cells were CD133-expressing (S2A Fig). These CD133^+^ cells were further isolated by magnetic beads and cultured in DMEM with highly concentrated growth factors EGF/bFGF. After 4 days culture, we observed that CD133^+^ cells generated typical spheres (S2D left Fig). Typical spheres were also generated from CD133^+^ PLC-PRF-5 cells (S10E Fig). In addition, CD133^+^ cells co-expressed higher levels of stem cell markers, such as SOX2, NANOG and OCT4, compared with that of CD133^-^ HepG2 cells (S2B Fig). These findings indicated that the HepG2 CD133^+^ cells were self-renewing hCSCs. Next, we examined and found the expression of RARs in CD133^+^ hCSCs was down-regulated (S3A–S3C Fig). For instance, the RARβ protein was lower expressed in CD133^+^ hCSCs than it was in CD133^-^ non-hCSCs (S3D Fig). Notably, we also detected that the expression level of the RARβ protein was much higher in HCC specimens than that in normal liver tissues (S3E Fig). However, no significant differences in the expression levels of RALDH1, ADH1 and RDH10 proteins were observed in HCC specimens compared with normal liver tissues (S10B Fig), with the exception of high ADH1 expression in normal liver tissue. Finally, we observed that the binding of ATRA (10^−9^ ~ 10^−5^ M) to RARs not only increased the mRNA expression of RARs (S4A–S4C Fig), but also directly enhanced their protein phosphorylation levels (S5 Fig).

### *All-trans* retinoic acid-induced differentiation of CD133^+^ hepatic cancer stem cells was dependent on β-catenin

**Fig 2:**
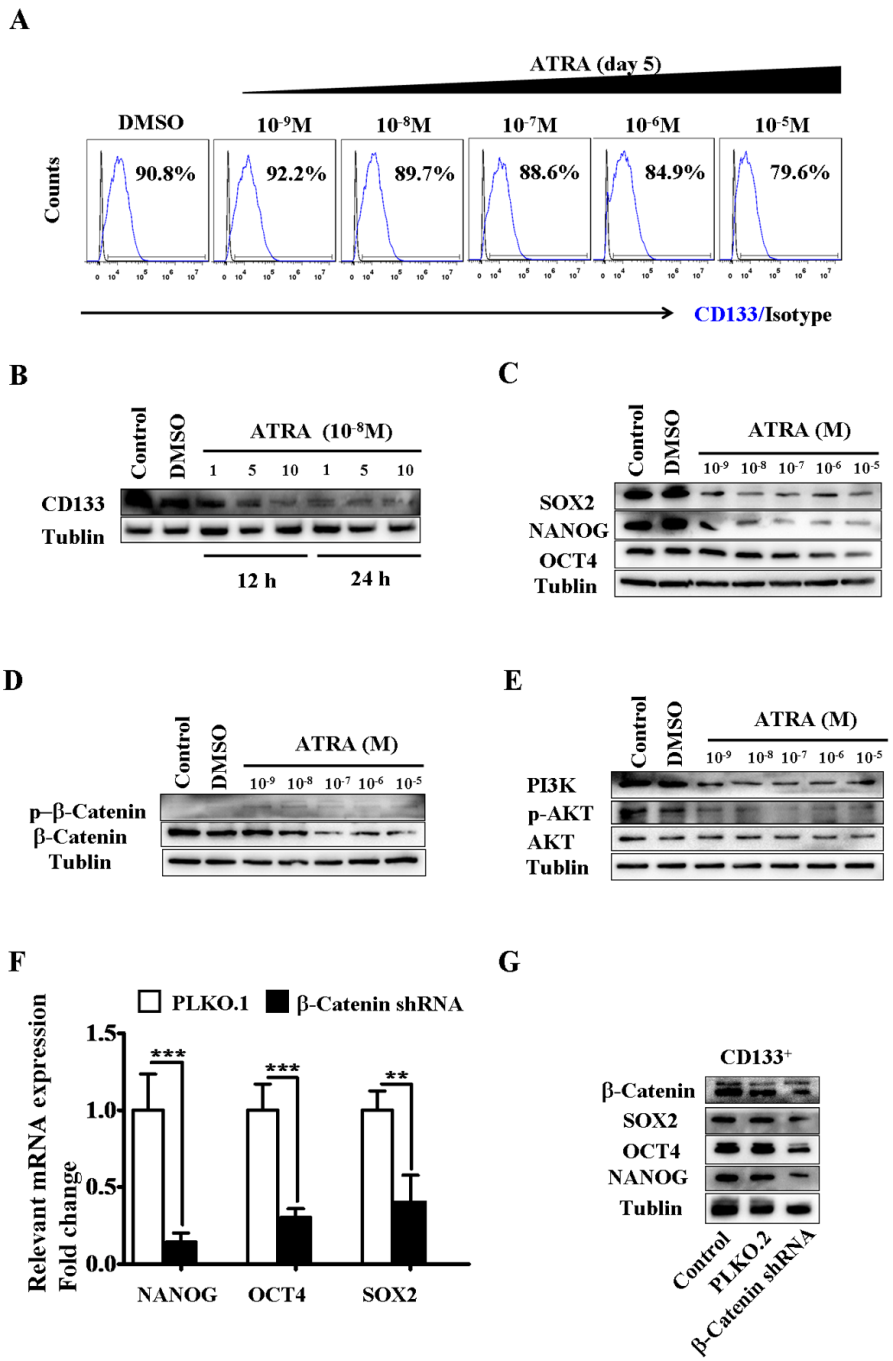
ATRA enhanced differentiation of CD133^+^ hCSCs by down-regulating the Wnt/β-catenin signaling pathway. (**A**) ATRA treatment (concentrations from 10^−9^ M to 10^−5^ M) decreased the percentage of CD133-expressing HepG2 cells at day 5 *in vitro*. (**B**) Expression of the CD133 protein decreased in CD133^+^ hCSCs after treatment with 1 × 10^−5^ M, 5 × 10^−5^ M and 10 × 10^−5^ M ATRA for 12 or 48 hours. *Control*, normal cell culture. (**C**) ATRA treatment down-regulated the protein expression level of other stem cell markers SOX2, NANOG and OCT4 after 5 days ATRA exposure (concentrations from 10^−9^ M to 10^−5^ M). (**D**) ATRA treatment decreased the protein expression level of β-catenin and increased its phosphorylation after 5 days ATRA exposure (concentrations from 10^−9^ M to 10^−5^ M). (**E**) ATRA inactivated the PI3K-AKT signaling pathway in CD133^+^ hCSCs after 5 days ATRA exposure (concentrations from 10^−9^ M to 10^−5^ M). (**F-G**) Silencing β-catenin mRNA decreased both the mRNA (**F**) and protein (**G**) expression of stem cell markers SOX2, NANOG and OCT4. *Control*, normal cell culture; *PLKO*.*1*, cells treated with empty vector PLKO.1; *β-catenin shRNA*, cells treated with the PLKO.1-β-catenin-shRNA constructed vector. ***p < 0.001; **p < 0.01.

Since RA is an endogenous differentiation signal, the effects of ATRA on the differentiation of CD133^+^ cells was further determined. We found that ATRA (10^−9^ ~ 10^−5^ M) decreased the number of CD133^+^ hCSCs in a concentration-dependent manner (Fig 2A). Consistently, we observed a significant down-regulation in stem cell marker proteins after ATRA treatment (Fig 2B and 2C). Similar results were investigated when 9-cis retinoic acid treated CD133^+^ hCSCs (S10A and S10C Fig). Furthermore, 9-cis retinoic acid treatment decreased the mRNA level in β-catenin (S10D Fig). These findings suggest that decreased CD133^+^ hCSCs were caused by the direct differentiation effect of ATRA. Interestingly, ATRA-induced differentiation of CD133^+^ hCSCs was associated with the activation of RARs (S4 and S5 Figs).

**Fig 3:**
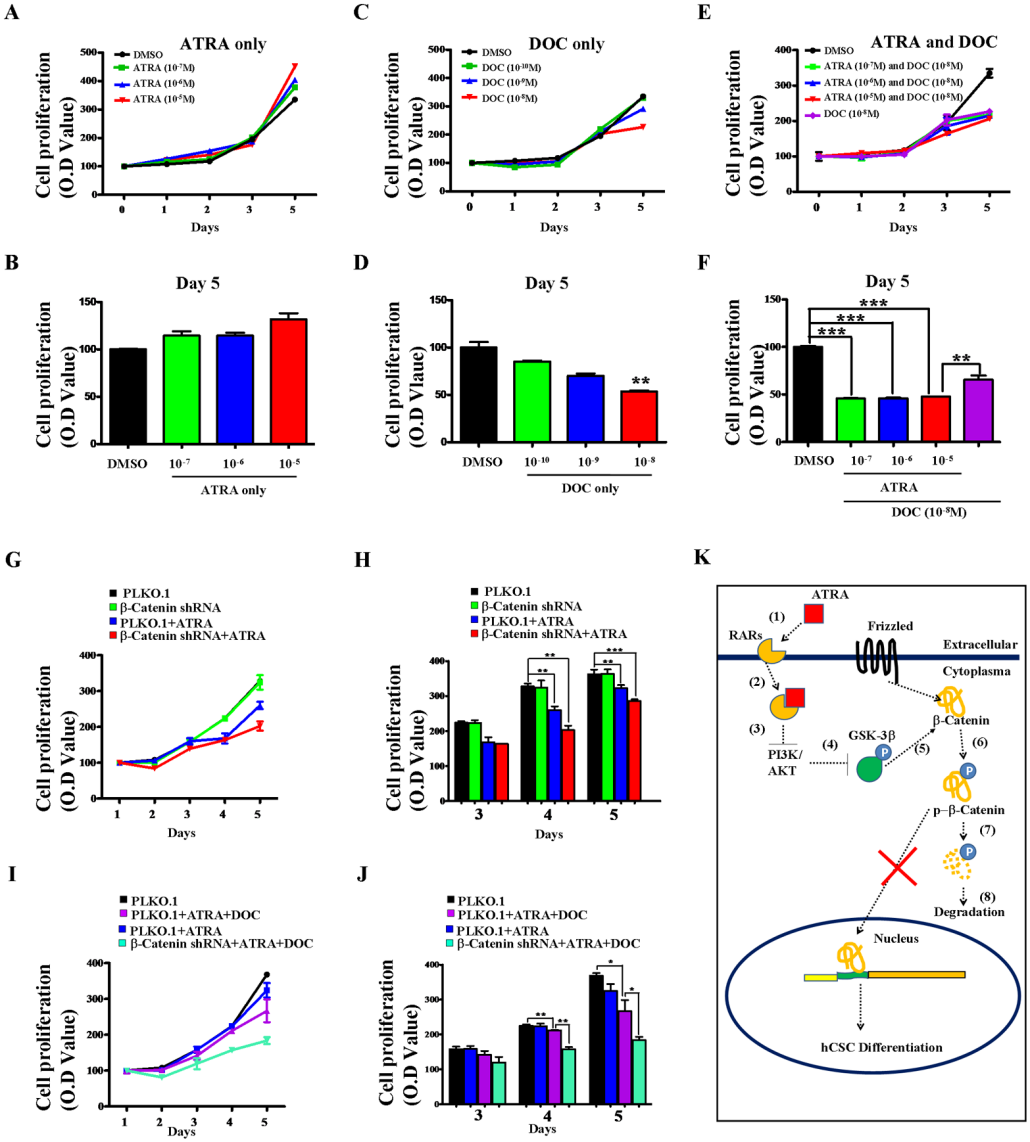
ATRA-induced differentiation of CD133^+^ hCSCs increased their sensitivity to docetaxel (DOC) treatment *in vitro*. (**A-B**) Survival and proliferation of CD133^+^ hCSCs after treatment with three concentrations (10^−5^ M, 10^−6^ M and 10^−7^ M) of ATRA. (**C-D**) Survival and proliferation of CD133^+^ hCSCs after treatment with three different concentrations (10^−10^ M, 10^−9^ M and 10^−8^ M) of DOC. (**E-F**) Combined treatment with ATRA (10^−5^ M, 10^−6^ M and 0^−7^ M) and DOC (10^−8^ M) decreased the growth of CD133^+^ hCSCs. (**A, B, C**) Growth curves of CD133^+^ hCSCs; (**D, E, F**) Quantitative analyses of CD133^+^ hCSC growth at day 5. ***p < 0.001; **p < 0.01. (**G-H**) ATRA treatment decreased the survival and proliferation of β-catenin mRNA knockdown-CD133^+^ hCSCs. (**I-J**) Knockdown of β-catenin mRNA decreased resistance of CD133^+^ hCSCs to DOC treatment (10^−8^ M). Concentration of ATRA in **G-J** is 10^−6^ M. (**G, I**) Growth curves of CD133^+^ hCSCs; (**H, J**) Quantitative analyses of CD133^+^ hCSC growth at day 3, day 4 and day 5. ***p < 0.001; **p < 0.01. (**K**) Molecular mechanism of ATRA-mediated differentiation of CD133^+^ hCSCs. Wnt/β-catenin was required to maintain the stemness of hCSCs. Binding of ATRA to RARs induced inactivation of the PI3K-AKT pathway, enhancing GSK-3β-dependent phosphorylation of β-catenin.

Previous studies suggested that ATRA inhibited cancer cell growth through down-regulating β-catenin protein expression (39–40). We hypothesized that ATRA-induced differentiation of CD133^+^ hCSCs was likely related to the function of β-catenin. We found that ATRA treatment (10^−9^ to 10^−5^ M) indeed decreased the protein level of β-catenin in CD133^+^ hCSCs (Fig 2D). At the same time, increased protein phosphorylation of β-catenin was observed after ATRA treatment (Fig 2D). It has been suggested that the Wnt/β-catenin signaling pathway is required to maintain the quiescent status of stem cells. Consistent with these findings, our results demonstrated that knockdown of β-catenin mRNA decreased the protein expression of stem cell markers in CD133^+^ hCSCs (Fig 2F and 2G) and impaired their stemness (S6A and S6B Fig). The regulation of β-catenin might be dependent on the PI3K-AKT pathway because ATRA decreased the protein level of PI3K and the protein phosphorylation of AKT (Fig 2E). The above findings support that ATRA-induced differentiation of CD133^+^ hCSCs was directly dependent on the function of β-catenin (Fig 3K).

### Differentiated CD133^+^ hepatic cancer stem cells were sensitive to docetaxel

We investigated whether ATRA-induced differentiation could provide a possible strategy to target CD133^+^ hCSCs. ATRA treatment (10^−7^ ~ 10^−5^ M) alone did not impact the survival or proliferation of CD133^+^ hCSCs (Fig 3A and 3B). Similarly, knockdown of β-catenin mRNA did not change the survival or proliferation of CD133^+^ hCSCs compared with that of the PLKO.1 vector control (Fig 3G and 3H, black: vector control, green: β-catenin shRNA). However, treatment with 10^−8^ M DOC decreased the survival and proliferation of CD133^+^ hCSCs due to apoptosis (S7A and S7B Fig). Compared with the single treatment of either ATRA or DOC, combined treatment with ATRA (10^−7^ ~ 10^−5^ M) and DOC (10^−8^ M) demonstrated much greater efficiency at decreasing CD133^+^ hCSC growth (Fig 3E and 3F). Consistently, we observed many more apoptotic CD133^+^ hCSCs in the combined treatments (S7A and S7B Fig). To confirm these results, we combined ATRA (10^−6^ M), β-catenin knockdown and DOC (10^−8^ M) and obtained an even more efficient targeting effect (Fig 3I and 3J). These results demonstrated that differentiation of CD133^+^ hCSCs increased their sensitivity to DOC treatment.

## Discussion

Although the liver is the only organ for retinoid metabolism, measurement of retinoid concentrations in liver cancer patients has not been reported. RA biosynthesis requires retinol dehydrogenases (RDH1, RDH10), alcohol dehydrogenases (ADH1, ADH2, ADH3) and retinaldehyde dehydrogenases (RALDH1, RALDH2, RALDH3) [35]. We analyzed the mRNA expression of the above dehydrogenases in HCC patients, and found that their mRNA expressions were the highest among 16 different cancers (Fig 1). The mRNA expressions of RALDH1 and RALDH2 were also slightly higher in HCC than in normal livers (S1 Fig), and protein expressions of RALDH1, ADH1 and especially RDH10 were also detected in hepatic carcinoma tissues (S10B Fig). To our best knowledge, this is the first time the activities of RA biosynthesis have been demonstrated in HCC patients, suggesting that RA could be enriched in the HCC microenvironment.

To determine whether RA is involved in the differentiation of hCSCs, we isolated self-renewing CD133^+^ hCSCs from the hepatocellular carcinoma cell line HepG2. We observed relatively low expressions of RARs and β-catenin in CD133^+^ hCSCs compared with those in CD133^-^ non-hCSCs (S2C, S3A and S3D Figs). Interestingly, binding of ATRA to RARs activated the phosphorylation of the receptors (S5 Fig). *In vitro*, ATRA directly differentiated CD133^+^ hCSCs, as indicated by the decreased protein expression of stem cell markers (Fig 2B and 2C). Further studies demonstrated that β-catenin was involved in ATRA-induced differentiation since its knockdown also decreased the protein expression of stem cell markers (Fig 2F and 2G). Another form of vitamin A, 9-cis retinoic acid, that activates both retinoid X and retinoic acid receptors, attenuated the mRNA expression of β-catenin and stem cell markers in hepatic CSCs (S10A, S10C and S10D Fig). Consistent with previous findings [39–40], we concluded that RA in HCC functions mainly as a differentiation signal for hCSCs. We found that ATRA induced the down-regulation of β-catenin protein expression, but increased the phosphorylation level of β-catenin (Fig 2D). Phosphorylated β-catenin can be further degraded though GSK3β [63] and the PI3K-AKT pathway may influence β-catenin phosphorylation by indirectly regulating the protein functions of GSK3β [57,59]. To verify this, we examined the effects of ATRA on the function of the PI3K-AKT pathway. Our results demonstrated that ATRA down-regulated the expression and phosphorylation of PI3K and AKT (Fig 2E). Therefore, we identified a potential RAR-mediated cytoplasmic signaling pathway that enhanced the GSK-3β-dependent degradation of β-catenin through the inactivation of the PI3K-AKT pathway (Fig 3K). In addition, our results also suggested that Wnt/β-catenin signaling was required to maintain the undifferentiated status of hCSCs.

ATRA has been clinically applied to treat cancer patients diagnosed with acute promyelocytic leukemia (APL) [63–65]. New clinical phase trials have also been conducted to develop ATRA as a potential anti-drug for other solid cancers [41,54–56]. However, we found that ATRA treatment alone did not obviously change cell survival or proliferation of CD133^+^ hCSCs (Fig 3A and 3B), with similar results also obtained when treating CD133^-^ non-hCSCs (S9 Fig). These findings suggest that solid hepatic cancer cells, in particular CD133^+^ hCSCs, may be resistant to ATRA treatment. Our results were consistent with previous studies in which lower drug levels of ATRA were clinically associated with relapses and ATRA-resistance in patients with APL [66]. This drug-related resistance might be because ATRA-induced differentiation needs molecular collaboration from other downstream pathways such as β-catenin. For instance, we found that ATRA-induced down-regulation of β-catenin increased hCSC sensitivity to docetaxel treatment (Fig 3E and 3F). Furthermore, combined inhibition of ATRA and β-catenin silencing also increased hCSC sensitivity to docetaxel treatment (Fig 3I and 3J).

Our research demonstrated that β-catenin potentially plays an essential role during ATRA-induced differentiation of hepatic cancer stem cells. Targeting β-catenin may provide extra benefits for hepatic cancer patients as the current treatment strategy mainly focuses on ATRA-mediated differentiation.

## Conclusions

Our results showed that ATRA differentiated hCSCs through a cytoplasmic signal pathway potentially composed of PI3K/Akt, GSK3β and β-catenin. β-catenin facilitated the undifferentiated status of hCSCs, suggesting that targeting β-catenin will provide extra benefits for ATRA-mediated treatment of hepatic cancer patients.

## Supporting Information

S1 Fig mRNA expressions of retinaldehyde dehydrogenases slightly increased in HCC. The mRNA expression profiling of three retinaldehyde dehydrogenases (RALDH1, RALDH2 and RALDH3) was conducted using two independent Oncomine data sets (Mas Liver Data set and Wurmbach Liver Data set). The Mas Liver Data set included 115 liver tissue samples separated into normal liver group (n = 19) and HCC (n = 38). The Wurmbach Liver Data set included 75 liver tissue samples classified into normal liver group (n = 10) and liver cell dysplasia (n = 17). The liver cancer group is highlighted in dark blue. (**A, B**) RALDH1; (**C, D**) RALDH2; (**E, F**) RALDH3; (**A, C, E**) Mas Liver Data set; (**B, D, F**) Wurmbach Liver Data set.
(TIF)

S2 Fig Isolation of CD133^+^ hCSCs. **(A)** Various percentages of CD133^+^ hCSCs were detected in three different human hepatic carcinoma cell lines by flow cytometry. HepG2 cell line (Left); Huh-7 cell line (Middle); PLC-PRF-5 cell line (Right). **(B-C)** Protein expressions of stem cell markers (**B**) and β-catenin (**C**) in both CD133^+^ and CD133^-^ subpopulations of HepG2 hepatic carcinoma cells. CD133^+^ hCSCs and CD133^-^ non-hCSCs are indicated by “-” and “+”, respectively. **(D)** CD133^+^ hCSCs were isolated from total HepG2 cells by magnetic beads and cultured to generate typical spheres. *CD133* ^+^ *hCSCs*, Sorted CD133-expressing HepG2 cells; *CD133* ^-^ *non-hCSCs*, HepG2 cells without CD133 expression.
(TIF)

S3 Fig RARs were differently expressed in CD133^+^ hCSCs and CD133^-^ non-hCSCs. (**A-C**) mRNA expressions of RARɑ, RARβ and RARγ were detected by qPCR in hCSCs and non-hCSCs. (**D**) Protein expression of RARβ in hCSCs and non-hCSCs. (**E**) Protein expression of RARβ in a HCC specimen diagnosed by pathological stage IV. RARβ-expressing cells are indicated by arrows.
(TIF)

S4 Fig ATRA increased mRNA expression of RARs in CD133^+^ hCSCs. mRNA expressions of RARɑ (**A**), RARβ (**B**) and RARγ (**C**) in CD133^+^ hCSCs treated with different concentrations (10^−9^ ~ 10^−5^ M) of ATRA. ***p < 0.001; **p < 0.01; *p < 0.05.
(TIF)

S5 Fig ATRA increased the protein expression and phosphorylation of RARs in CD133^+^ hCSCs. (TIF)

S6 Fig Knockdown of β-catenin mRNA expression in CD133^+^ hCSCs. (**A**) Five different lentiviral shRNA constructs of β-catenin were initially screened to determine their knockdown efficiency. *Control*, normal cell culture; *PLKO*.*1*, empty lentiviral shRNA vector; *#1-#5*, five different lentiviral β-catenin shRNA constructs. (**B**) *In vitro* growth of CD133^+^ hCSC-derived tumorspheres after β-catenin knockdown by #4 construct. Tumorspheres are indicated by arrows. Scale bar, 200 μm.
(TIF)

S7 Fig Combined treatment with both ATRA and DOC induced apoptosis of CD133^+^ hCSCs. (**A-B**) Apoptosis in CD133^+^ hCSCs treated with ATRA (10^−7^ M), DOC (10^−9^ M) or ATRA (10^−7^ M)/DOC (10^−9^ M). *P1*, percentage of CD133^+^ hCSCs in all events. *P3*, *P4* and *P6* represent late apoptotic, early apoptotic and non-apoptotic cell populations, respectively.
(TIF)

S8 Fig Gene expression profiling of β-catenin in different cancer types. (**A**) Yu multi-cancer data set (341 clinical samples). Category #4 represents HCC group (9 clinical samples); (**B**) Su multi-cancer data set (174 clinical samples). Category #6 represents HCC group (7 clinical samples). HCC specimens are highlighted by red boxes.
(TIF)

S9 Fig Effect of ATRA on survival and growth of CD133^-^ non-hCSCs. (**A**) Survival and proliferation of CD133^**-**^ non-hCSCs after treatment with three concentrations (10^−5^ M, 10^−6^ M and 10^−7^ M) of ATRA. (**B**) Quantitative analysis of CD133^**-**^ non-hCSC survival and growth after 5 days treatment with ATRA.
(TIF)

S10 Fig Effect of 9-cis RA on CD133^+^ hCSCs and expressions of RALDH1, ADH1 and RDH10 in hepatic carcinoma. (**A**) Flow cytometry analysis of cellular membrane CD133 level after 5 days 9-cis retinoic acid exposure (10^−7^ M, 10^−6^ M and 10^−5^ M). (**B**) Protein expression of RALDH1, RDH10 and ADH1 in a HCC specimen diagnosed by pathological stage IV. RALDH1, RDH10 and ADH1-expressing cells are indicated by arrows. (**C**) mRNA level of stem cell markers NANOG, OCT4 and SOX2 after 5 days 9-cis retinoic acid exposure (10^−7^ M, 10^−6^ M and 10^−5^ M). (**D**) mRNA level of β-catenin after 5 days 9-cis retinoic acid exposure (10^−7^ M, 10^−6^ M and 10^−5^ M). (**E**) CD133^+^ hCSCs were isolated from total PLC-PRF-5 cells by magnetic beads and cultured to generate typical spheres. *CD133* ^+^ *hCSCs*, Sorted CD133-expressing PLC-PRF-5 cells; *CD133* ^-^ *non-hCSCs*, PLC-PRF-5 cells without CD133 expression.
(TIF)

## Abbreviations

RA: retinoic acid
ATRA: *all-trans* retinoic acid
CSCs: cancer stem cells
CICs: cancer-initiating cells
hCSCs: hepatic cancer stem cells
HCC: hepatocellular carcinoma
RDH: retinol dehydrogenases
ADH: alcohol dehydrogenases
RALDH: retinaldehyde dehydrogenases
DOC: docetaxel
RAREs: retinoic acid elements
