# Supplementary figures and images for: All-Trans Retinoic Acid-Induced Deficiency of the Wnt/β-Catenin Pathway Enhances Hepatic Carcinoma Stem Cell Differentiation
Source: PLoS One. 2015 Nov 16;10(11):e0143255. doi: 10.1371/journal.pone.0143255 (PMC4646487; doi:10.1371/journal.pone.0143255)

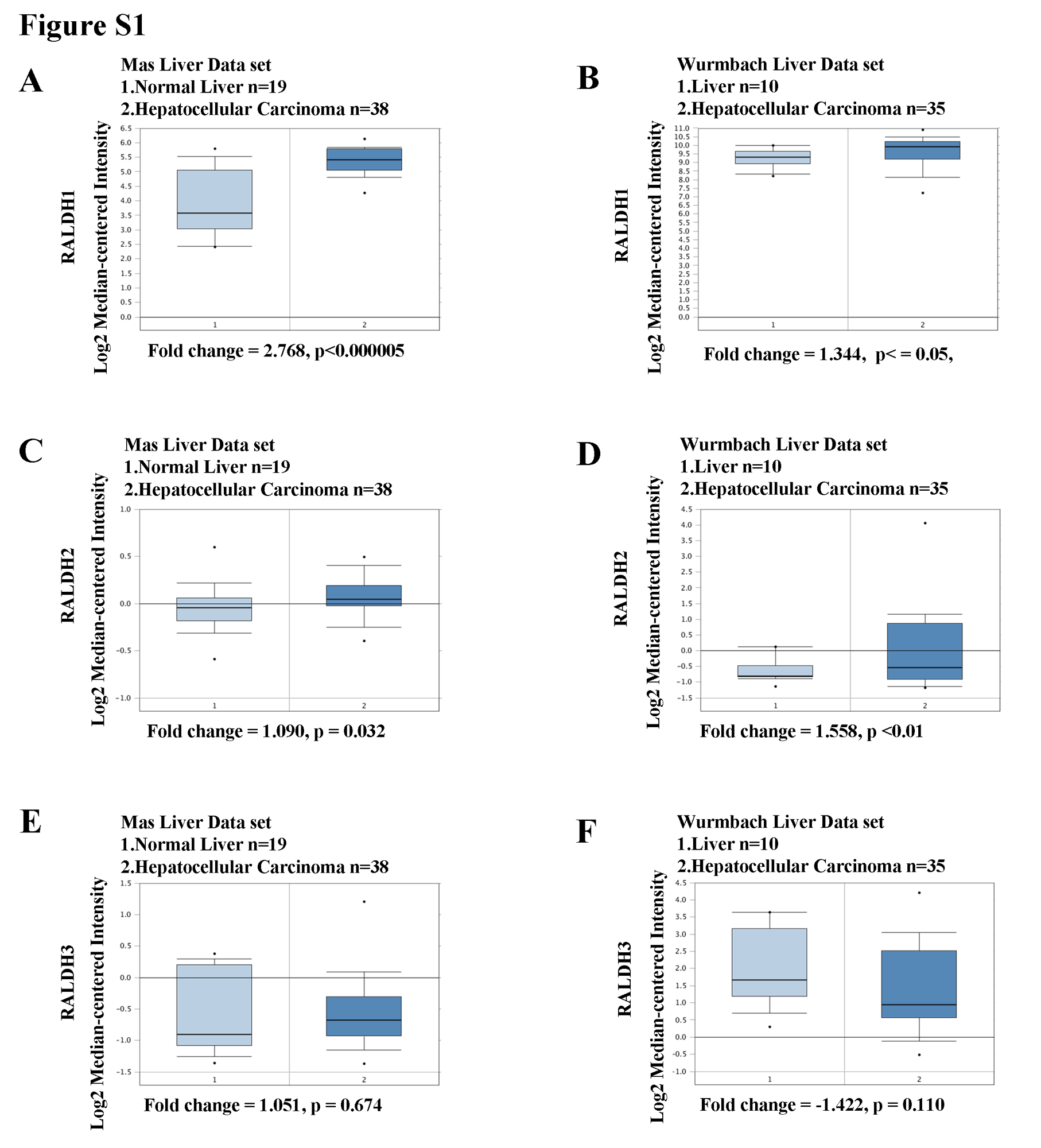

Supplement: S1 Fig — The mRNA expression profiling of three retinaldehyde dehydrogenases (RALDH1, RALDH2 and RALDH3) was conducted using two independent Oncomine data sets (Mas Liver Data set and Wurmbach Liver Data set). The Mas Liver Data set included 115 liver tissue samples separated into normal liver group (n = 19) and HCC (n = 38). The Wurmbach Liver Data set included 75 liver tissue samples classified into normal liver group (n = 10) and liver cell dysplasia (n = 17). The liver cancer group is highlighted in dark blue. (A, B) RALDH1; (C, D) RALDH2; (E, F) RALDH3; (A, C, E) Mas Liver Data set; (B, D, F) Wurmbach Liver Data set. (TIF) [file pone.0143255.s001.tif]

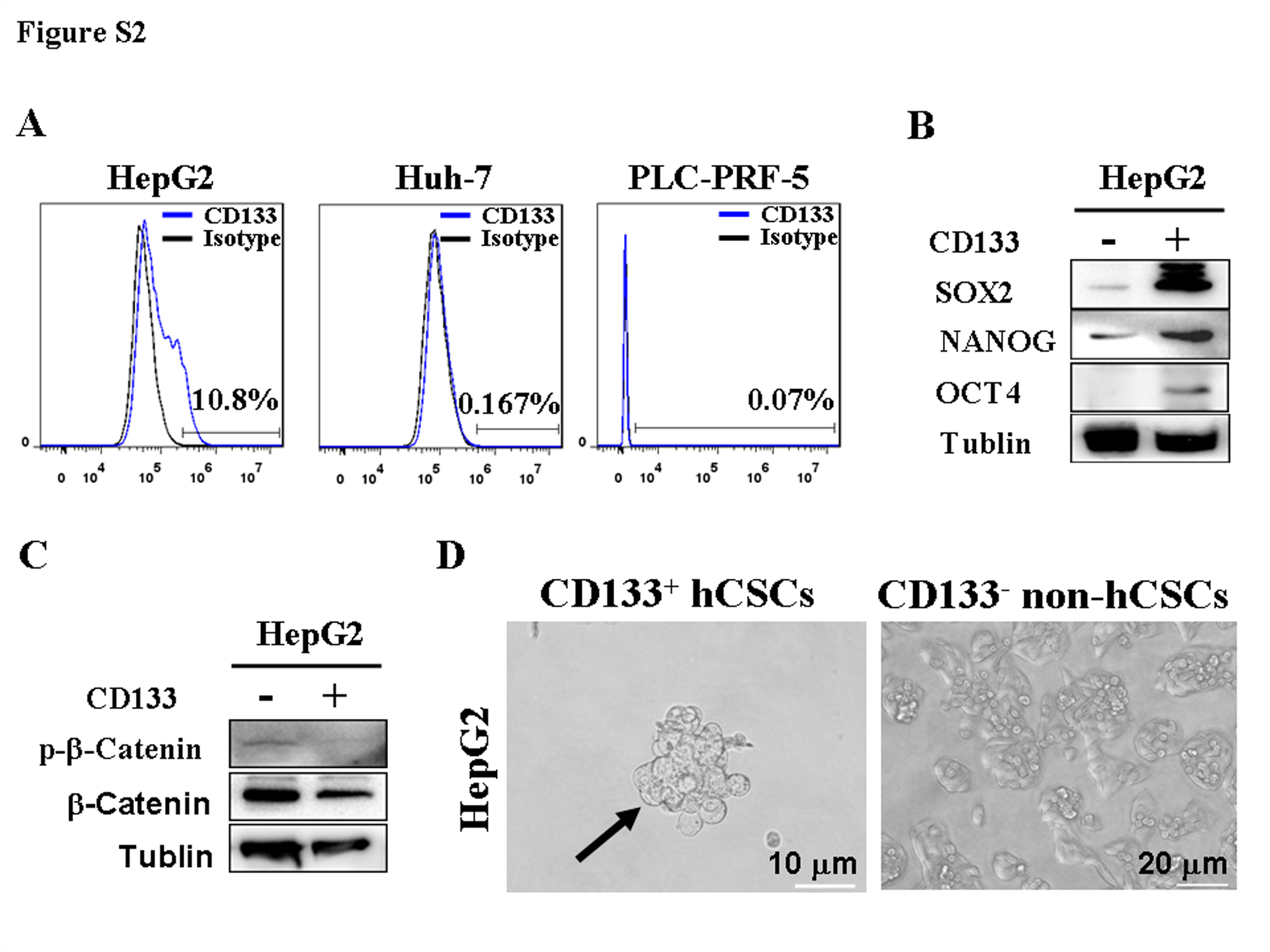

Supplement: S2 Fig — (A) Various percentages of CD133+ hCSCs were detected in three different human hepatic carcinoma cell lines by flow cytometry. HepG2 cell line (Left); Huh-7 cell line (Middle); PLC-PRF-5 cell line (Right). (B-C) Protein expressions of stem cell markers (B) and β-catenin (C) in both CD133+ and CD133- subpopulations of HepG2 hepatic carcinoma cells. CD133+ hCSCs and CD133- non-hCSCs are indicated by “-” and “+”, respectively. (D) CD133+ hCSCs were isolated from total HepG2 cells by magnetic beads and cultured to generate typical spheres. CD133 + hCSCs, Sorted CD133-expressing HepG2 cells; CD133 - non-hCSCs, HepG2 cells without CD133 expression. (TIF) [file pone.0143255.s002.tif]

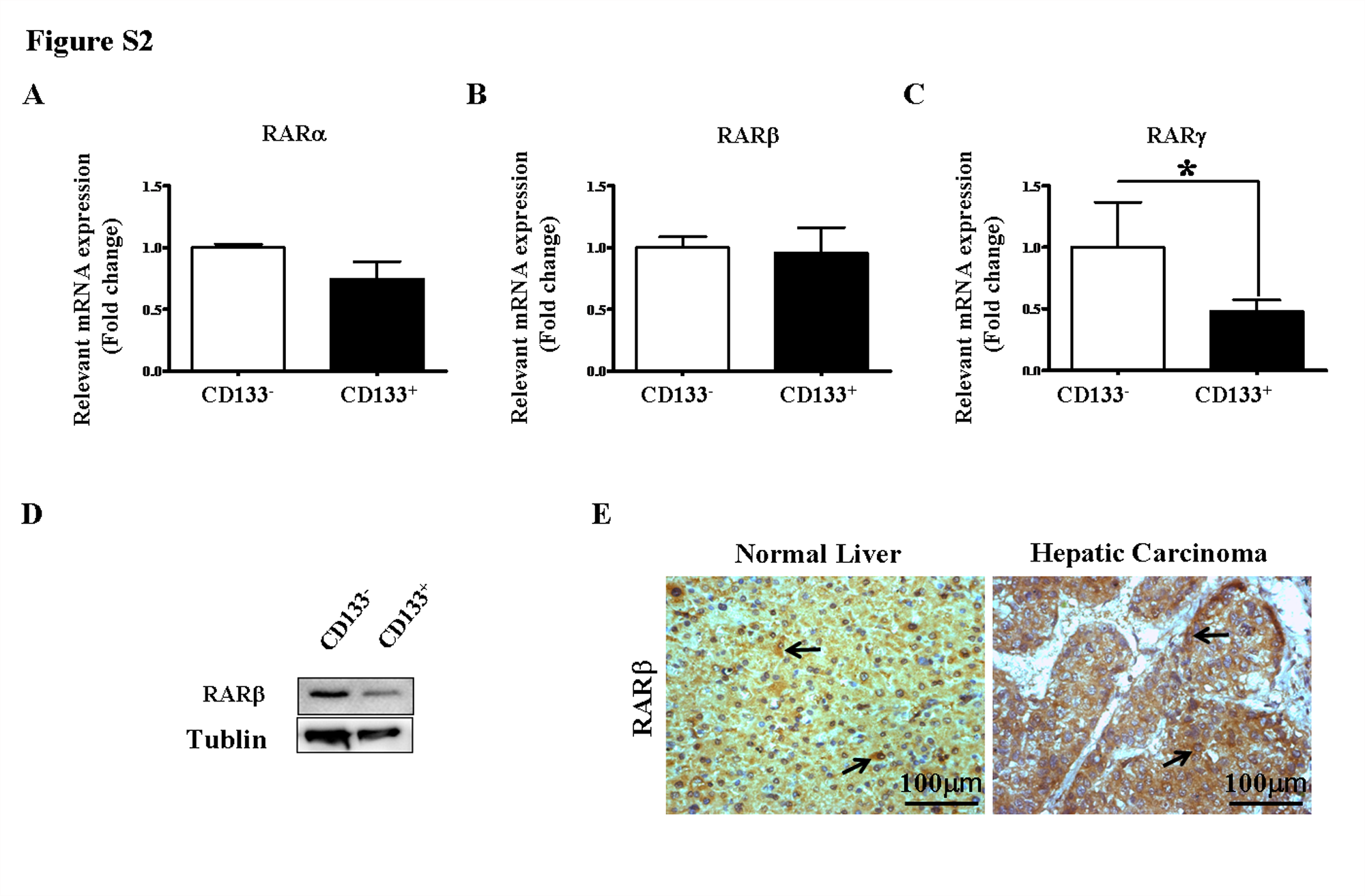

Supplement: S3 Fig — (A-C) mRNA expressions of RARɑ, RARβ and RARγ were detected by qPCR in hCSCs and non-hCSCs. (D) Protein expression of RARβ in hCSCs and non-hCSCs. (E) Protein expression of RARβ in a HCC specimen diagnosed by pathological stage IV. RARβ-expressing cells are indicated by arrows. (TIF) [file pone.0143255.s003.tif]

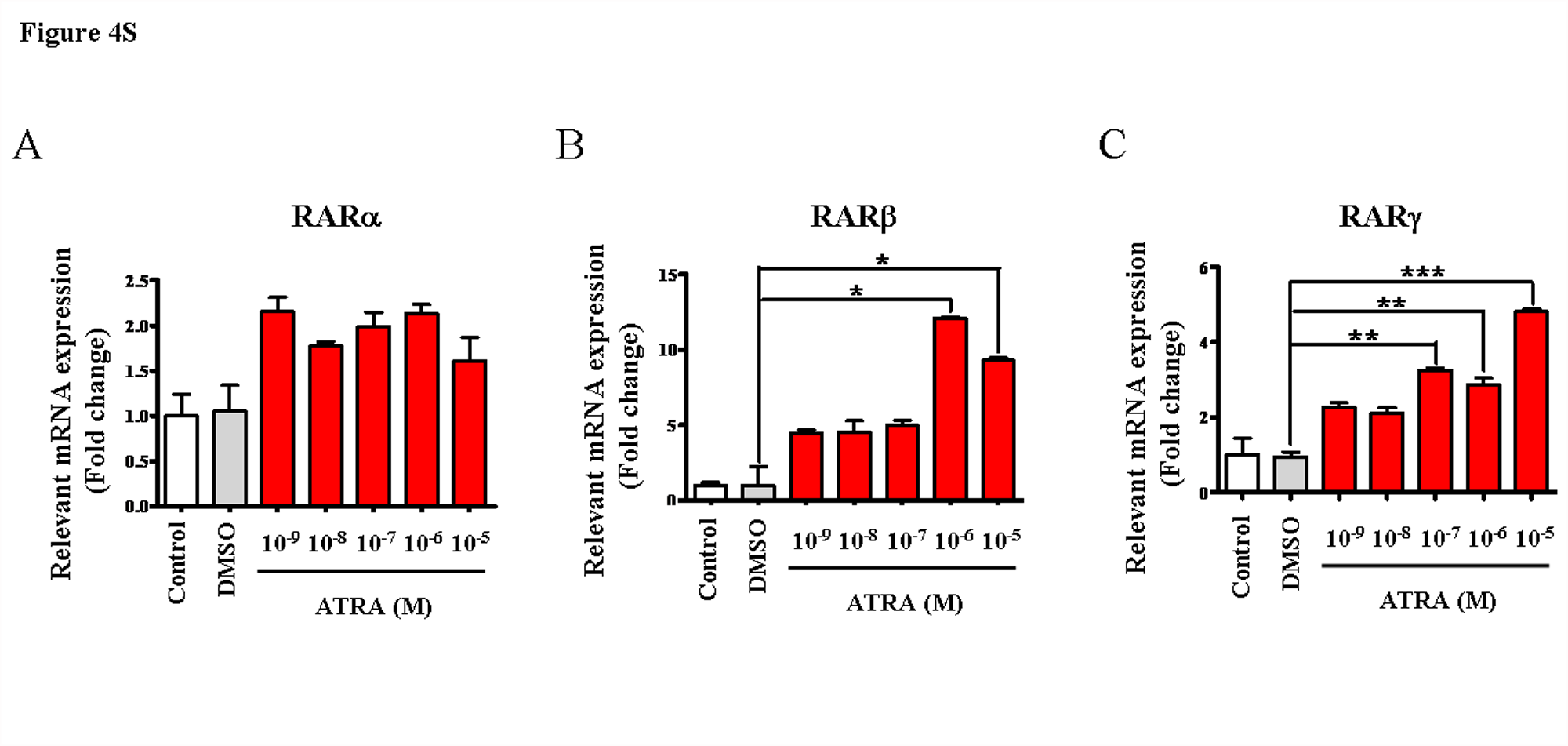

Supplement: S4 Fig — mRNA expressions of RARɑ (A), RARβ (B) and RARγ (C) in CD133+ hCSCs treated with different concentrations (10−9 ~ 10−5 M) of ATRA. ***p < 0.001; **p < 0.01; *p < 0.05. (TIF) [file pone.0143255.s004.tif]

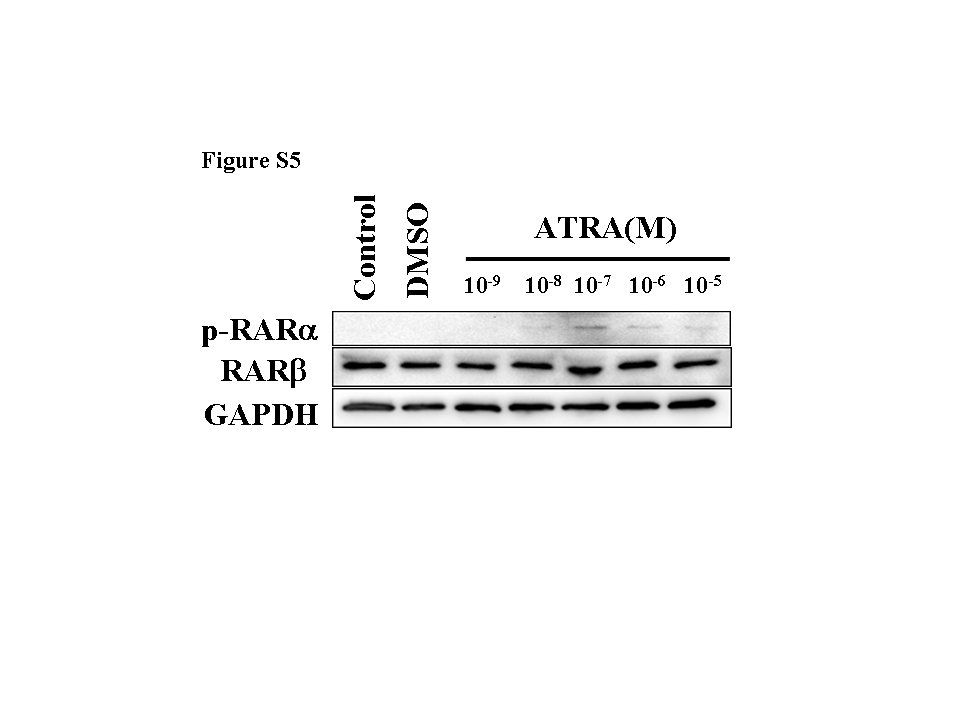

Supplement: S5 Fig — (TIF) [file pone.0143255.s005.tif]

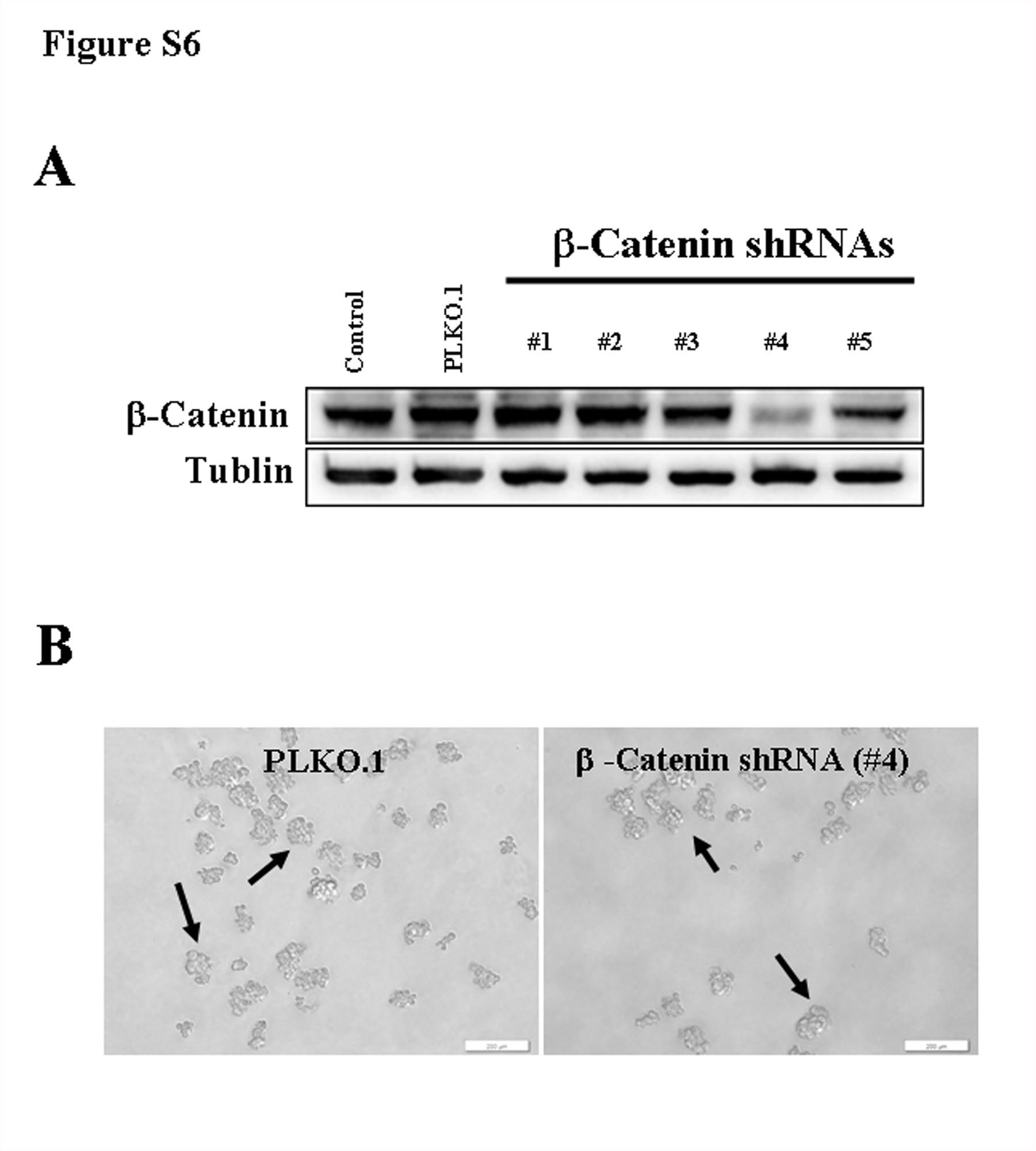

Supplement: S6 Fig — (A) Five different lentiviral shRNA constructs of β-catenin were initially screened to determine their knockdown efficiency. Control, normal cell culture; PLKO.1, empty lentiviral shRNA vector; #1-#5, five different lentiviral β-catenin shRNA constructs. (B) In vitro growth of CD133+ hCSC-derived tumorspheres after β-catenin knockdown by #4 construct. Tumorspheres are indicated by arrows. Scale bar, 200 μm. (TIF) [file pone.0143255.s006.tif]

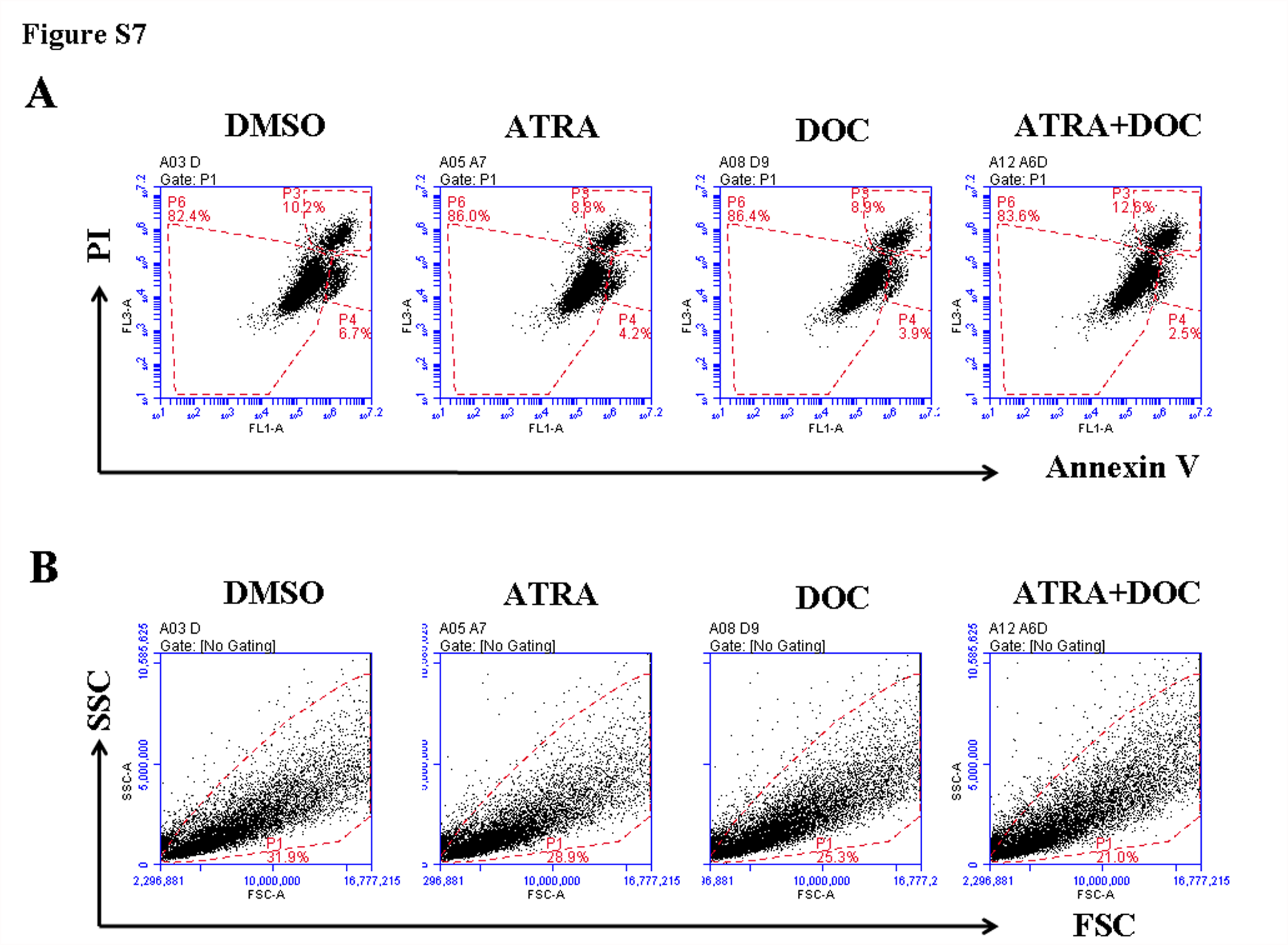

Supplement: S7 Fig — (A-B) Apoptosis in CD133+ hCSCs treated with ATRA (10−7 M), DOC (10−9 M) or ATRA (10−7 M)/DOC (10−9 M). P1, percentage of CD133+ hCSCs in all events. P3, P4 and P6 represent late apoptotic, early apoptotic and non-apoptotic cell populations, respectively. (TIF) [file pone.0143255.s007.tif]

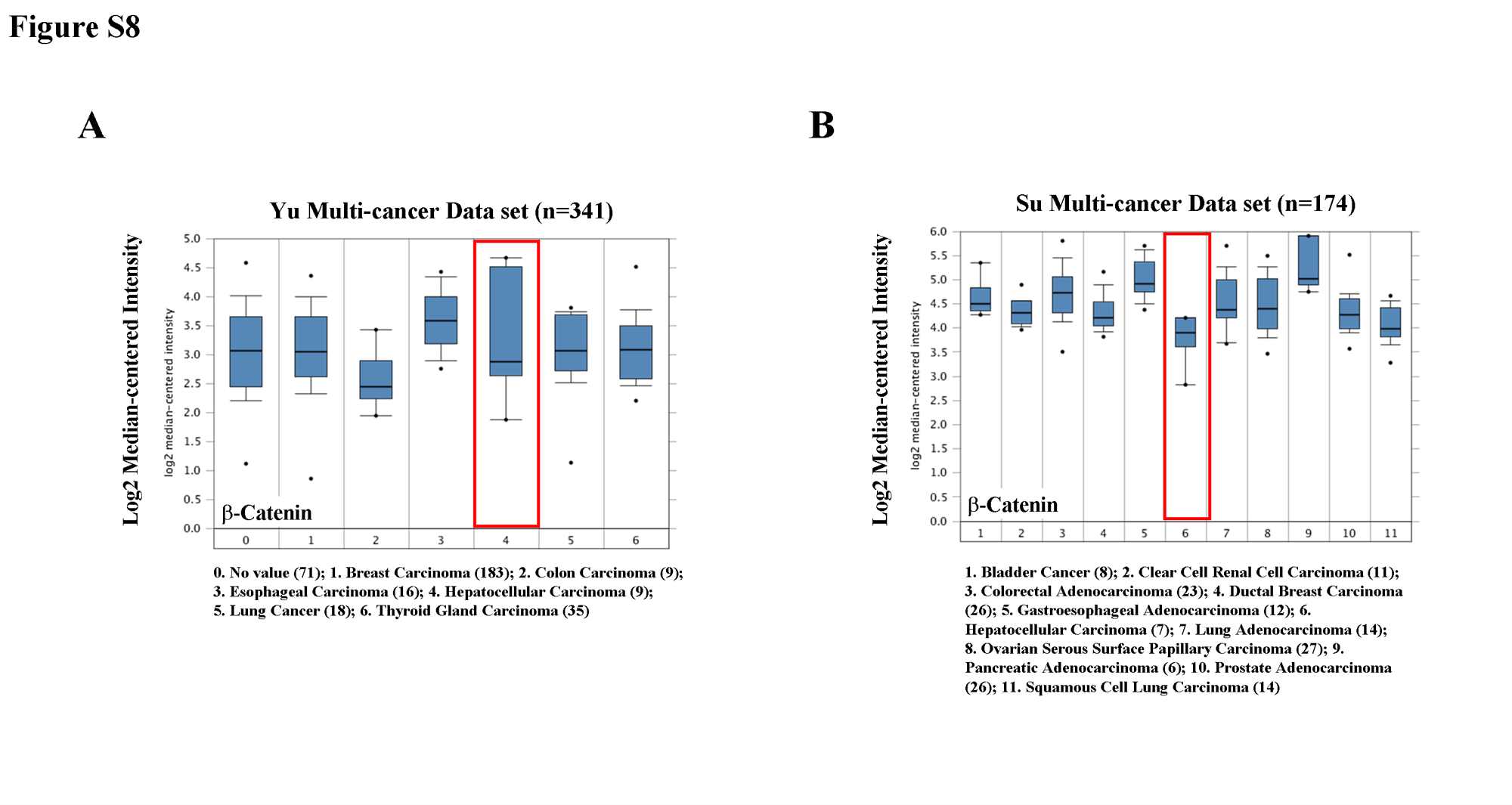

Supplement: S8 Fig — (A) Yu multi-cancer data set (341 clinical samples). Category #4 represents HCC group (9 clinical samples); (B) Su multi-cancer data set (174 clinical samples). Category #6 represents HCC group (7 clinical samples). HCC specimens are highlighted by red boxes. (TIF) [file pone.0143255.s008.tif]

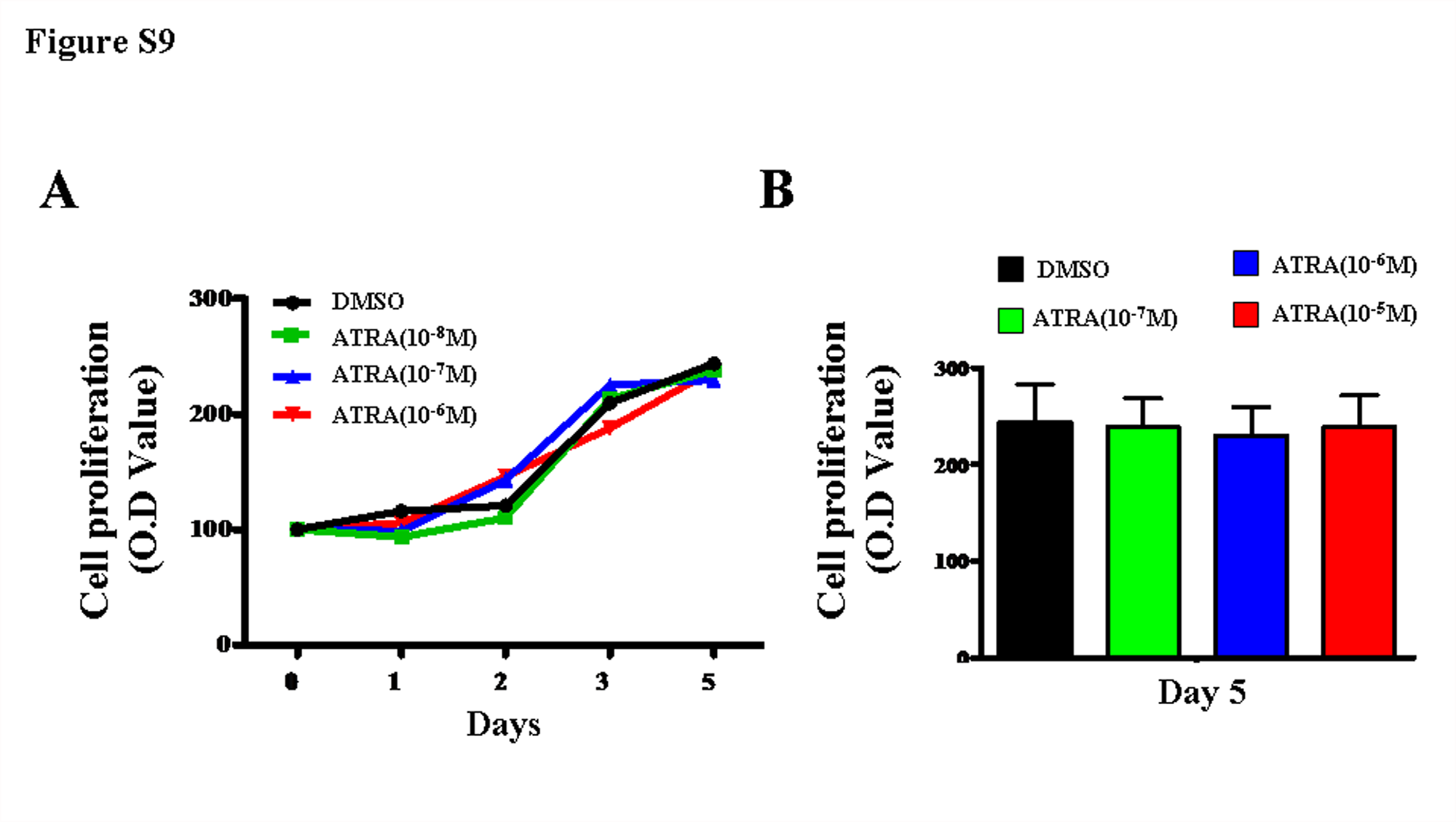

Supplement: S9 Fig — (A) Survival and proliferation of CD133- non-hCSCs after treatment with three concentrations (10−5 M, 10−6 M and 10−7 M) of ATRA. (B) Quantitative analysis of CD133- non-hCSC survival and growth after 5 days treatment with ATRA. (TIF) [file pone.0143255.s009.tif]

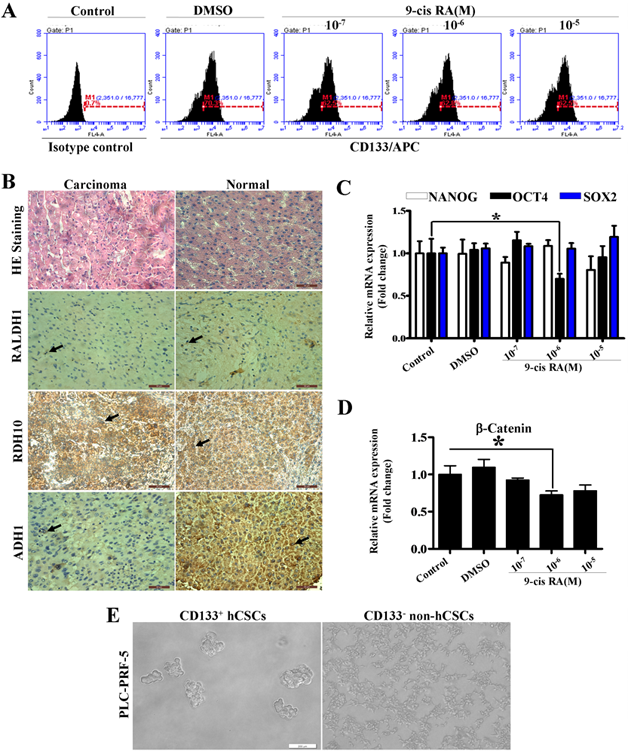

Supplement: S10 Fig — (A) Flow cytometry analysis of cellular membrane CD133 level after 5 days 9-cis retinoic acid exposure (10−7 M, 10−6 M and 10−5 M). (B) Protein expression of RALDH1, RDH10 and ADH1 in a HCC specimen diagnosed by pathological stage IV. RALDH1, RDH10 and ADH1-expressing cells are indicated by arrows. (C) mRNA level of stem cell markers NANOG, OCT4 and SOX2 after 5 days 9-cis retinoic acid exposure (10−7 M, 10−6 M and 10−5 M). (D) mRNA level of β-catenin after 5 days 9-cis retinoic acid exposure (10−7 M, 10−6 M and 10−5 M). (E) CD133+ hCSCs were isolated from total PLC-PRF-5 cells by magnetic beads and cultured to generate typical spheres. CD133 + hCSCs, Sorted CD133-expressing PLC-PRF-5 cells; CD133 - non-hCSCs, PLC-PRF-5 cells without CD133 expression. (TIF) [file pone.0143255.s010.tif]
